# A Diagnostically Challenging Parotid Gland Tumor With Hybrid Features

**DOI:** 10.7759/cureus.15383

**Published:** 2021-06-02

**Authors:** Steven Hamilton, Maleeha Saleem, Mustafa Ali, Adam C Kaplan, Gopi Mukkavilli

**Affiliations:** 1 Internal Medicine, Jersey Shore University Medical Center/Saint Francis Medical Center Program, Trenton, USA

**Keywords:** pleomorphic adenoma, biphasic tumor, fine needle aspiration cytology (fnac), mucoepidermoid carcinoma, enucleation, enucleoresection

## Abstract

Salivary gland tumors are relatively uncommon with most being benign. When diagnosed the most common benign and malignant tumors are pleomorphic adenoma and mucoepidermoid carcinoma (MEC), respectively. However, not uncommonly, it is difficult to differentiate between the histopathological entities, leading to a diagnostic dilemma that can impact a patient’s treatment and prognosis. A 24-year-old woman presented with a three-year history of asymptomatic left-sided facial swelling. She denied any prior history of head and neck radiation. There was no history of alcohol consumption or smoking exposure and there was no personal or family history of head and neck cancers. Additionally, she did not have any known occupational or environmental exposures. Due to the chronicity and painless nature of this facial mass, our patient did not pursue evaluation initially. Subsequently, she experienced an increase in size and pain for a few months exacerbated by swallowing. She had no other symptoms. On physical examination, a 3 x 3 cm left parotid gland mass was noted. There was no associated head or neck lymphadenopathy and compression of the left facial mass did not elicit secretions from the opening of Stensen’s duct. Due to the rapid increase in size, she was sent for CT neck/soft tissue with contrast which confirmed a 3.56 x 2.67 cm solid nodule within the superficial portion of the left parotid gland. This was followed by an MRI orbit/face/neck with and without contrast, for further delineation, which demonstrated a 4 x 3.7 x 3 cm complex heterogeneous mass within the superficial left parotid gland. Thereafter the patient underwent an uncomplicated ultrasound-guided biopsy of the parotid mass. The histopathological appraisal concluded that this was a cellular pleomorphic adenoma, with mucinous and squamous metaplasia with reactive lymph nodes. Due to the new rapid increase in size and intense painful nature of this tumor, nerve-sparing left parotidectomy, fat grafting and reconstruction were completed. Cellular pleomorphic adenomas are benign low-grade neoplasms, typified as biphasic with both epithelial and myoepithelial components. However, they have increased cellularity and focally increased mitotic activity, not advanced enough to qualify as malignant. The presence of mucinous and squamous metaplasia is of diagnostic interest as it makes diagnosis on fine-needle aspiration (FNA) morphologically challenging. These findings are typical of MEC and on FNA can be misleading in the setting of a pleomorphic adenoma. However, on histopathological evaluation of the gross specimen along with immunohistochemical staining the diagnosis is made much easier. A diagnosis of MEC would have potentially required neck dissection and adjuvant therapy with a potential increased risk of morbidity and mortality. This case emphasises the importance of an adequate tissue biopsy in regards to parotid gland tumors to optimise a patient's care plan.

## Introduction

Salivary gland tumors are rare, making up less than 5% of all head and neck neoplasms [1]. The majority are benign with the parotid gland being the most common hub for these tumors (85%) and the remainder occurring in the submandibular and minor salivary glands in subsequently lower rates of 5% and 10%, respectively [2]. Pleomorphic adenomas or benign mixed salivary gland tumors are the most common benign tumors of the parotid gland, as well as the minor salivary glands [3]. These tumors may occur at any age however the most commonly affected age group is between 30 and 50 years of age, with a slight female predominance (2:1). The annual incidence is roughly 2 to 3.5 cases per 100,000 population, which demonstrates its rarity. The key distinguishing features involving varying combinations of epithelial and myoepithelial cells in a mucinous or stromal background in different histological subtypes of pleomorphic adenoma forms the basis of identification and the distinction from mucoepidermoid carcinoma (MEC) is equally important as the latter has a totally different management in regards to surgery and follow up. It is a slow-growing tumor and most individuals present late primarily due to increase in size and facial disfigurement [4-6]. These indolent large tumors are most frequently treated by facial nerve-sparing total parotidectomy but malignant transformation in an indolent tumor must be excluded with negative surgical margins [7]. The recommended post-operative surveillance period for these patients is at least three to four years [8]. The recurrence rate of pleomorphic adenomas is high, with the recurrent tumors frequently lacking a capsule, which presents a surgical challenge with respect to resection. We are reporting a case that represents a diagnostic challenge in terms of the histopathological appraisal of both the fine-needle aspiration cytology (FNAC) specimen and the surgically resected gross specimen.

## Case presentation

A 24-year-old woman who immigrated from Liberia to the USA approximately one year ago presented our outpatient clinic with a complaint of a left-sided facial swelling for the past three years. She initially noticed this left lower jaw swelling approximately three years ago but left it untreated due to the chronic nature of the swelling and her relatively asymptomatic course. However, subsequently, she noticed an interval increase in the size of the swelling associated with pain. This was aggravated by palpation of the swelling, lying on her left side and swallowing. There was no associated fever, drainage from the swelling, increased salivary production, anorexia, weight loss, dyspnea, dysphagia, hoarseness, ophthalmoplegia, hearing loss, gait disturbance, or known tuberculosis exposure. She did not endorse smoking, alcohol consumption or use illicit drugs. There was no personal or family history of cancers. Additionally, she did not have any known occupational or environmental exposures. This was on the background of having no known medical history of any chronic illness.

**Figure 1:**
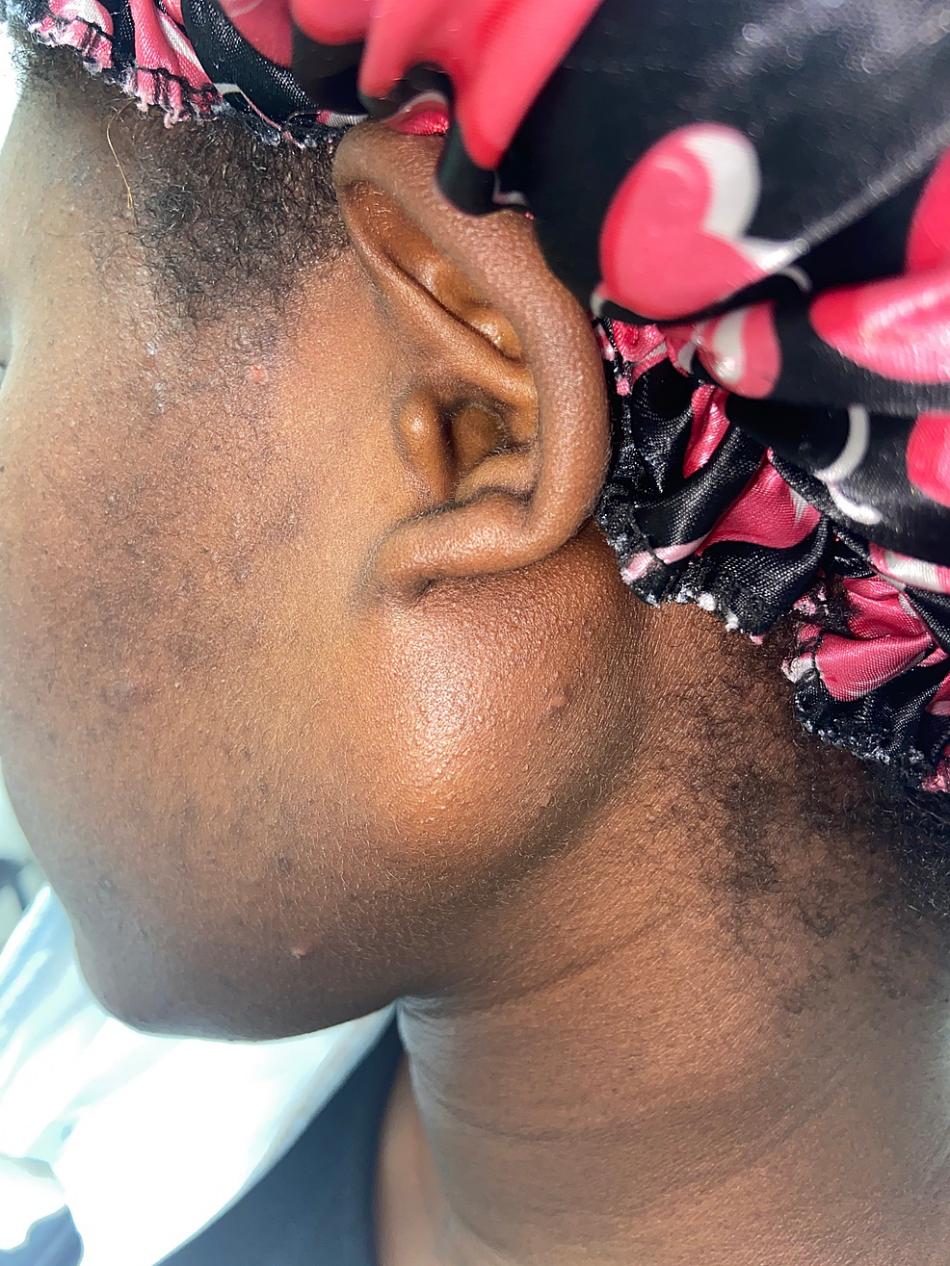
Pre-operative photograph demonstrating the left parotid gland swelling.

The patient was well developed with vital signs within normal limits. On physical examination, there was a 3 x 3 cm, mildly tender, non-mobile, round, firm, non-compressible and non-reducible swelling at the angle of mandible abutting the lobule of the left ear (Figure 1). No cervical, supraclavicular or axillary lymphadenopathy was appreciated and there were no overlying skin changes. There was no associated facial asymmetry or loss of sensation. On examination of the oral cavity, the swelling was not palpable internally. Compression of the left facial mass did not elicit secretions from the opening of Stensen’s duct.

**Figure 2:**
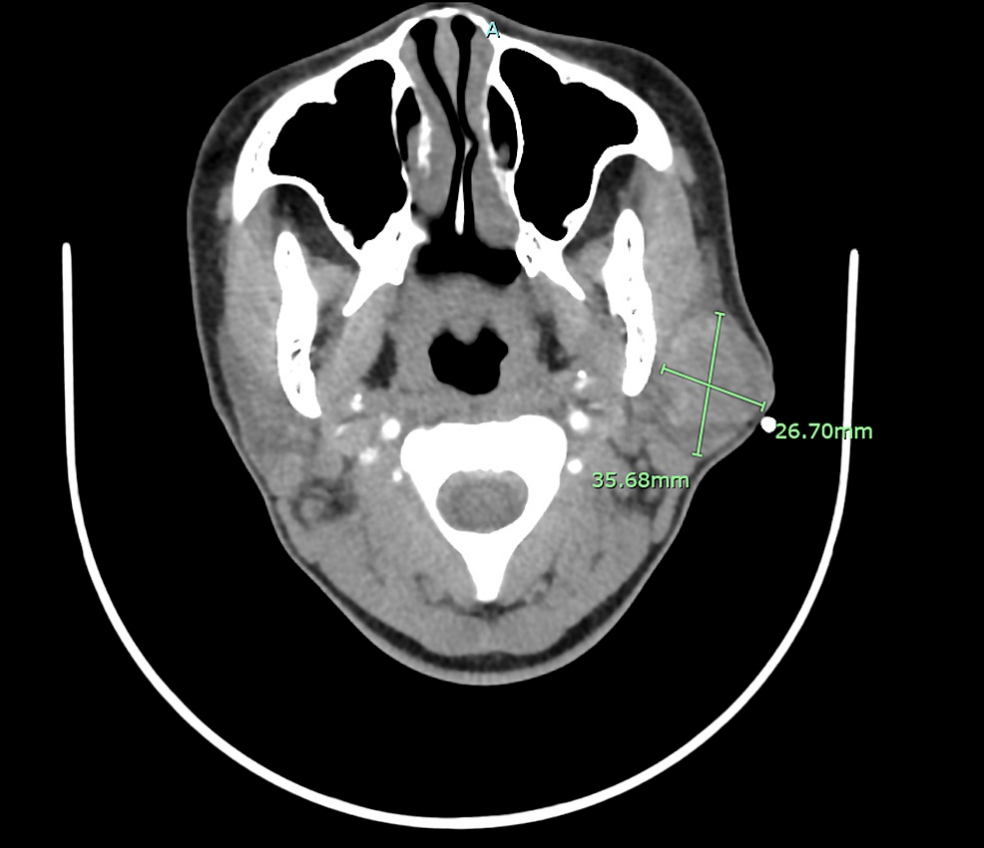
CT scan of soft tissue of neck with contrast demonstrating a solid nodule in the superficial portion left parotid gland measuring 3.56 x 2.67 cm that appeared to be homogeneous and had a well-defined margin.

**Figure 3:**
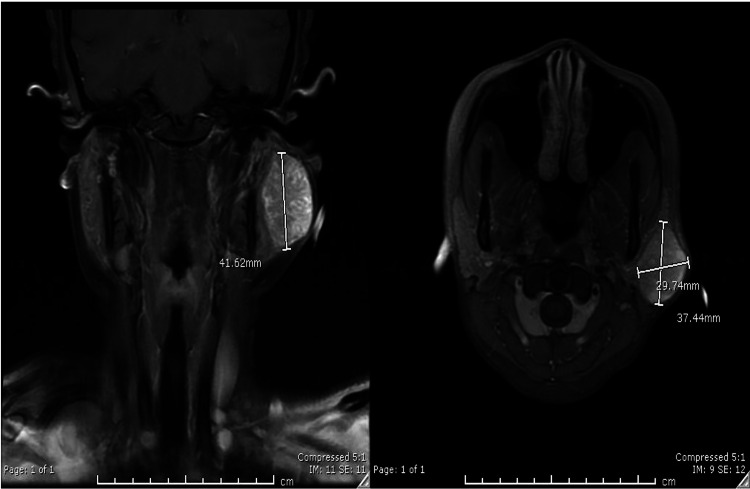
MRI orbit/face/neck without contrast which revealed an indeterminate complex heterogeneous enhancing mass within the superficial left parotid gland.

**Figure 4:**
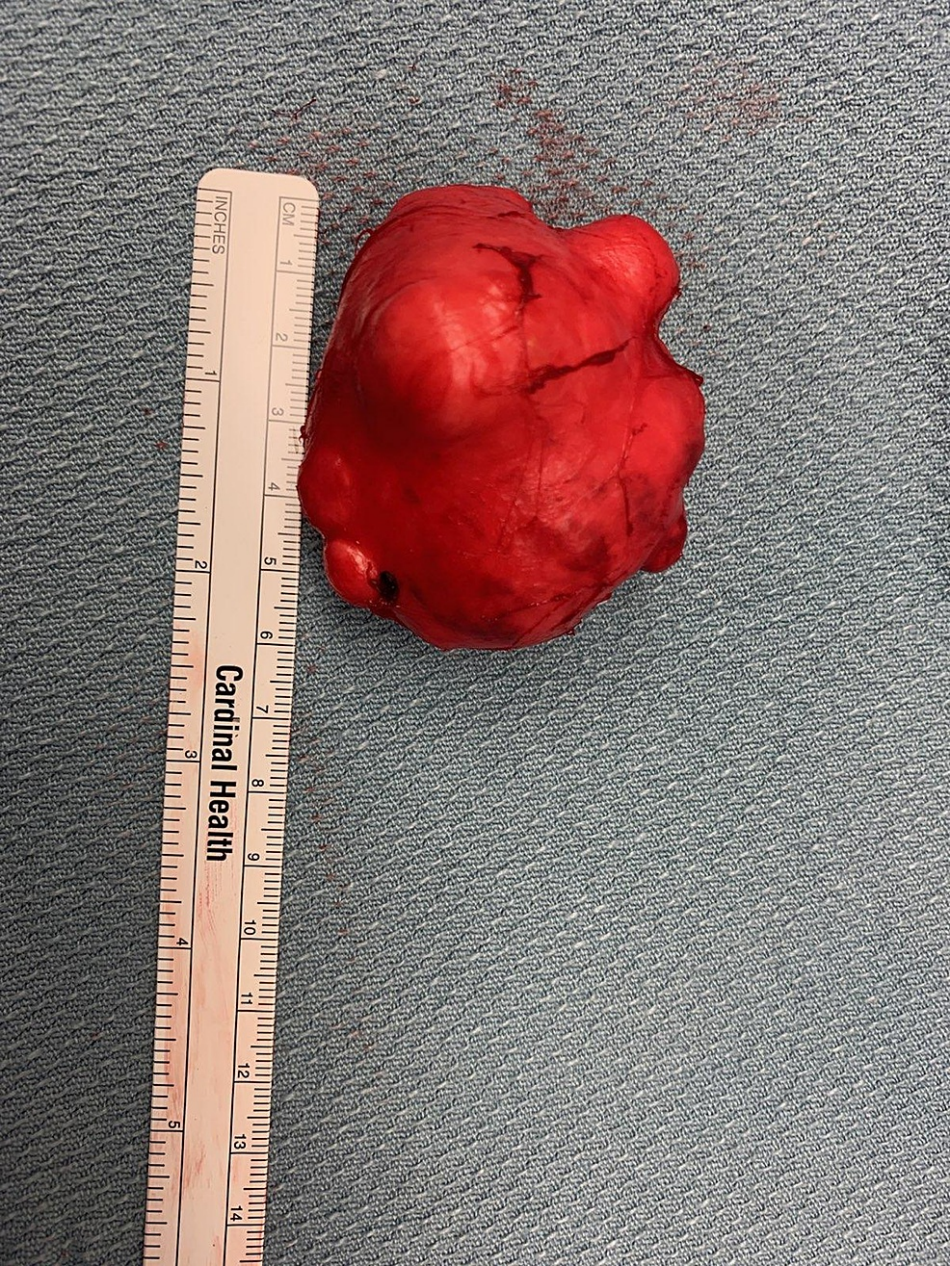
The gross specimen of the resected left parotid gland tumor.

**Figure 5:**
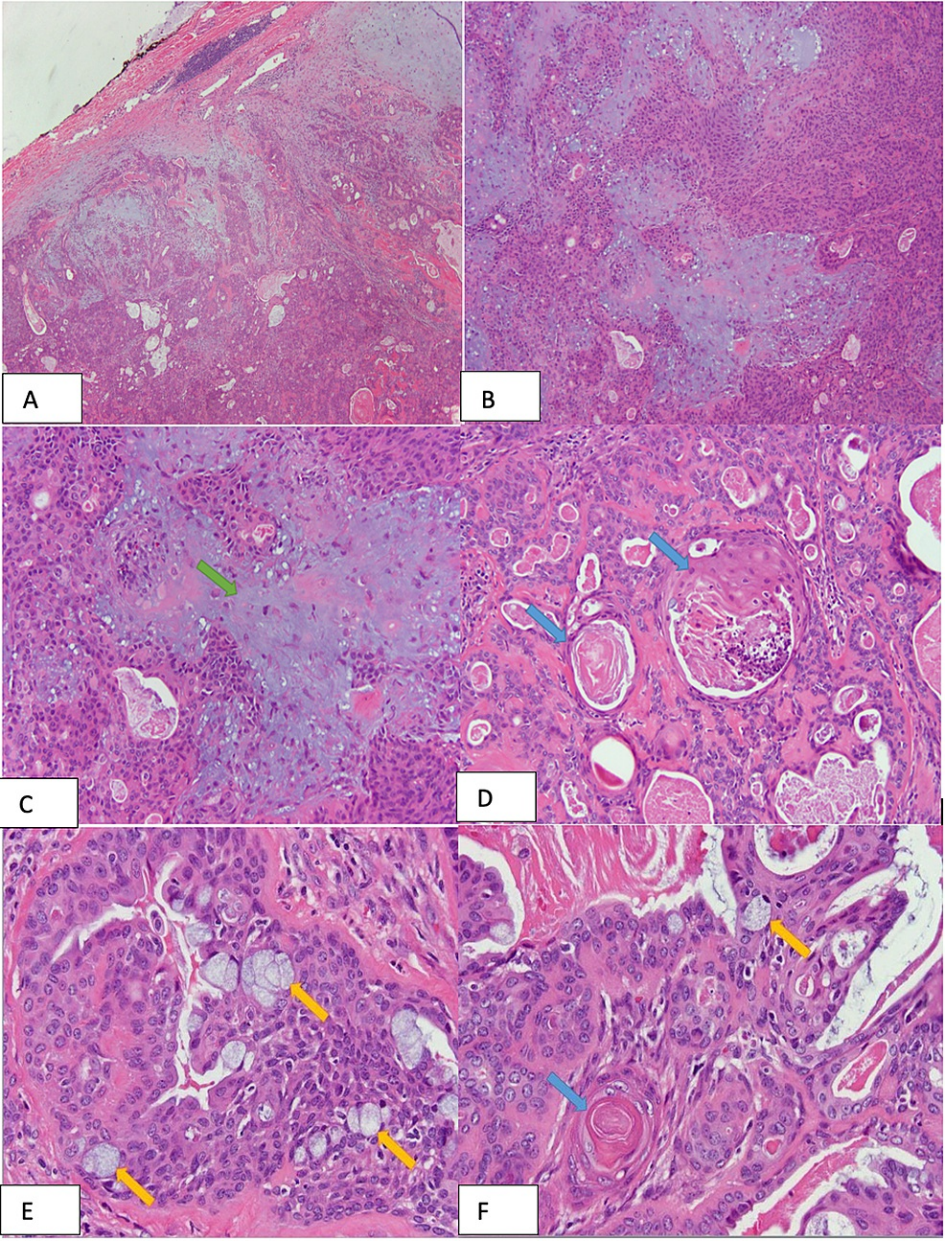
Photomicrographs demonstrating the pertinent histologic findings. (A) The tumor contained a well-circumscribed border (4x). (B) Classic features of pleomorphic adenoma were identified, including a biphasic epithelial cell (ductal and myoepithelial) population and a chondromyxoid stroma. The stromal component occupied only a minor proportion of the tumor, hence the diagnosis of a cellular pleomorphic adenoma. (10x). (C) This field shows the characteristic chondromyxoid stroma (green arrow) embedded with myoepithelial cells, typical of a pleomorphic adenoma (20x). (D) The ductal cells exhibited areas of squamous metaplasia (blue arrows) (20x). (E) Foci of mucinous metaplasia (yellow arrows) were also identified (40x). (F) This field contains both squamous (blue arrow) and mucinous (yellow arrow) metaplasia of the ductal cells (40x).

**Figure 6:**
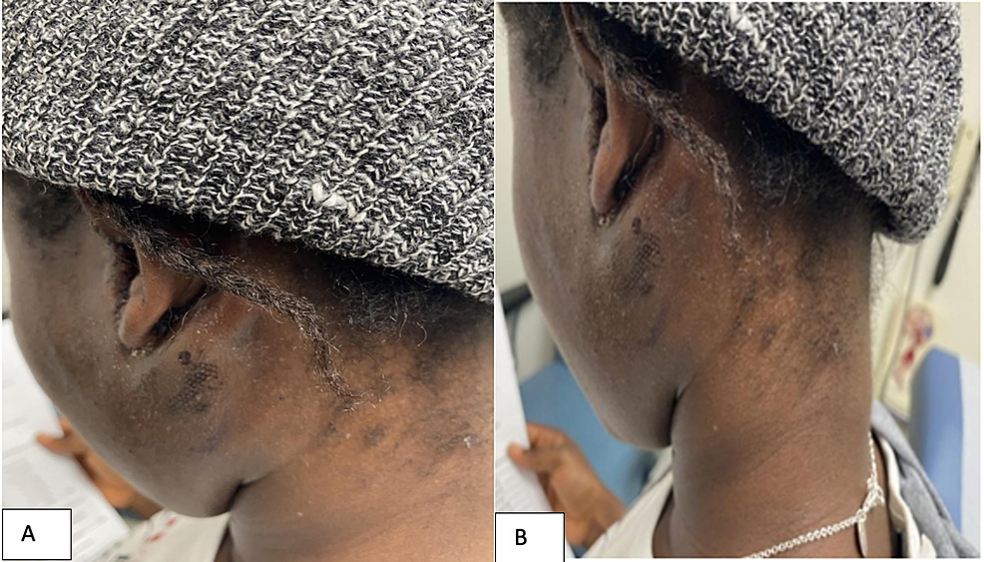
A, B: Post-operative photographs.

Due to the rapid increase in size and a concern for possible malignancy, the patient was sent for a CT scan of soft tissue of neck with contrast that confirmed a solid nodule in the superficial portion left parotid gland measuring 3.56 x 2.67 cm that appeared to be homogeneous and had a well-defined margin (Figure 2). The right parotid gland, thyroid gland and submandibular glands were unremarkable. There was no evidence for prevertebral soft tissue swelling. Thereafter, in order to further delineate the lesion, she underwent MRI orbit/face/neck without contrast which revealed an indeterminate complex heterogeneous enhancing mass within the superficial left parotid gland. The mass measured 4 x 2.7 x 3 cm (Figure 3). There was no evidence of cervical lymphadenopathy and the patient was scheduled for ultrasound-guided percutaneous core needle biopsy of the left parotid mass as this has increased sensitivity compared to FNAC. The post-resection specimen consisted of approximately six cores of tan tissue ranging from 1.3 to 1.5 cm in length x 0.1 cm in width. The FNAC report revealed a salivary gland neoplasm with squamous and mucinous differentiation, with further classification not being possible. Due to the new rapid increase in size and painful nature of mass, she was scheduled for surgery and underwent nerve-sparing left parotidectomy (Figure 4), fat grafting and reconstruction. The final histopathological appraisal concluded that this was a cellular pleomorphic adenoma (Figure 5), with mucinous and squamous differentiation with reactive lymph nodes. She has since been followed in the outpatient setting where the surgical site is healing well without any facial nerve abnormalities (Figure 6).

 

 

## Discussion

Cellular pleomorphic adenomas are benign low-grade neoplasms, typified as biphasic with both epithelial and myoepithelial components [4]. In this content, the lesion is characterized by several different histological subtypes including myxoid, chondroid and mucoid while remaining a benign tumor [9,10]. Morphologically, pleomorphic adenomas have a false capsule, which is the compressed salivary gland giving the appearance of the false capsule. This tumor grows as finger-like extensions into the normal salivary gland [11]. Due to the relatively indolent growth pattern of the tumor, many patients have a delayed presentation, with facial disfigurement or local symptoms due to mass effect [6].

On gross evaluation, the majority of these tumors measure between 2 and 4 cm [12]. Facial nerve involvement is rare and only occurs in very large tumors and more commonly in malignant lesions like mucoepidermoid carcinoma (MEC). Our patient's main presentation was because of a rapid increase in the size of tumor and disabling localized pain. Fine-needle aspiration cytology (FNAC) is the most commonly used diagnostic modality. Tumors may be biphasic as mentioned earlier containing epithelial cells and chondromyxoid stroma in varying proportions. However, rarely they may display features such as squamous metaplasia and mucinous metaplasia. This suggests increased cellularity and focally increased mitotic activity, not advanced enough to qualify as malignant. The presence of mucinous and squamous metaplasia is of diagnostic interest as it makes diagnosis on fine-needle aspiration (FNA) morphologically challenging. The aforementioned findings are typical of neoplastic or reactive conditions of the salivary glands, such as chronic sialadenitis, radiation change of salivary glands and necrotizing sialometaplasia, MEC, pleomorphic adenoma, basal cell adenoma and Warthin's tumor. The presence of these features on FNAC can be misleading in the presence of a pleomorphic adenoma [13] as was present in our case, as further classification was not possible on the sample. Subsequently, it brought distress to the patient and a diagnostic dilemma to the clinical team as it was particularly challenging to exclude a malignancy of the parotid gland via FNAC.

Imaging modalities such as CT scan and MRI assist in staging the pleomorphic adenoma. MRI is superior to CT Scan as it informs about the depth of involvement, surrounding structures including bone and the neurovascular bundle that can assist in guiding surgery. With respect to other diagnostic modalities, an incisional biopsy is contraindicated as it leads to an increased chance of recurrence [5]. We acknowledge that serologies such as Epstein-Barr virus may be helpful in determining an etiology but unfortunately, this was not available for our patient.

Histopathologically, MEC is classified into low, intermediate and high grade, with low grade (48%) being more common than high grade (38.7%) and intermediate grade (13.3%) being the least common. The degree of cytological atypia, cyst formation and relative numbers of mucous, epidermoid and intermediate cells are the determinants of tumor grade [1,14]. Some pleomorphic adenomas may exhibit malignant potential and the incidence of malignant transformation has been reported. The risk is high in long-standing cases, very large tumors, recurrences, submandibular location, hyalinised connective tissue and older age of the patient [15]. That being said, carcinoma ex pleomorphic adenoma is extremely rare [16]. Immunohistochemical studies for our patient revealed the tumor to be positive for p63 and CAM5.2, two markers with high specificity for pleomorphic adenoma. p63 is a marker of myoepithelial differentiation that is absent in carcinoma ex pleomorphic adenoma and present in pleomorphic adenoma and CAM 5.2 is a marker for epithelial cell differentiation that is commonly positive in pleomorphic adenoma. Positivity for both markers suggested a biphasic nature of this pleomorphic adenoma [17,18]. The relative contribution of cellular and stromal composition defines whether a pleomorphic adenoma is predominantly cellular or stromal. In the case of our patient, there was more of the epithelial component and less of the chondromyxoid stroma, hence the classification as cellular pleomorphic adenoma. Histologically, a cartilaginous, mucoid or myxoid stromal background is mostly encountered in pleomorphic adenoma.

Subsequent to the inconclusive FNAC report revealing squamous and mucinous metaplasia and staging imaging not showing regional or distant disease, the patient was scheduled to undergo surgery. Thereafter she underwent a superficial parotidectomy with excision of the left parotid mass and two reactive lymph nodes. The histopathological evaluation of the gross specimen with squamous and mucinous metaplasia was not advanced enough to be classified as malignant. There was the absence of atypia, metastasis, tumor necrosis and the presence of minimal cellular proliferative activity in the context of a slow-growing nature of tumor. It was important to make the most accurate diagnosis as labelling the tumor as MEC would have potentially required neck dissection and adjuvant therapy with potential increased risk of morbidity and mortality. Nerve-sparing enucleoresection and superficial parotidectomy formed the mainstay of surgical treatment. Generally, treatment depends upon tumor size, location and extent of local structure involvement [19]. Extreme care should be taken during surgery to achieve nerve-sparing resection and avoid tumor spillage in order to reduce morbidity and recurrence with increased mortality respectively. Our patient had an uncomplicated post-surgical period. Follow-up revealed a healing surgical site and no symptoms/signs suggestive of recurrence. In keeping with current recommendations, our patient will be followed for signs of recurrence for at least three years.

## Conclusions

Salivary gland tumors are uncommon. When they do occur, they may present as a diagnostic challenge necessitating a multidisciplinary approach for tissue sampling and eventual appraisal. It is imperative to make the correct diagnosis as this determines management course. Combined clinicopathological assessment is the recommended approach as either in isolation may yield equivocal results. This case highlights the importance of obtaining an adequate tissue biopsy for appropriate histopathological appraisal in order to guide subsequent management of the tumor. 
